# Loneliness and Trust: An East-West Comparison in Europe

**DOI:** 10.1093/geroni/igaa057.2062

**Published:** 2020-12-16

**Authors:** Marja Aartsen, Gražina Rapolienė

**Affiliations:** 1 OsloMet Oslo Metropolitan University, Oslo, Norway; 2 Lithuanian Social Research Centre, Vilnius, Vilniaus Apskritis, Lithuania

## Abstract

Loneliness in later life is two times more prevalent in Eastern and Southern European countries than in Northern and Western European countries. One explanation that is put forth is the difference in expectations about social relations. We examine a not often evaluated role of trust in society as factor contributing to the country differences in loneliness. We adopt the trust-as-antecedent model of social integration, and assume that social integration is associated with loneliness. We use data of respondents aged 65 and over participating in the European Social Survey and conduct a latent factors path analysis to examine the effect of trust in the system and trust in people on social capital and loneliness. Loneliness is two times more prevalent in Eastern Europe than the rest of Europe (26% vs 10%), levels of trust are substantially lower in Eastern European countries, which in turn is associated with higher levels of loneliness.

